# Evaluation of Different Cryoprotectant Combinations in Testicular Vitrification in Dogs

**DOI:** 10.1111/rda.70081

**Published:** 2025-05-30

**Authors:** Jéssyka Araújo Noronha, Juliana de Souza Fernandes, Francisco Denilson Rodrigues Gomes, Julia Carrah Colares, Gisele Karla Sena Guimarães, Herlon Victor Rodrigues Silva, Lúcia Daniel Machado da Silva

**Affiliations:** ^1^ Laboratório de Reprodução de Carnívoros (LRC), Universidade Estadual do Ceará (UECE) Fortaleza CE Brazil; ^2^ Laboratório de Manipulação de Oócitos e Folículos Ovarianos Pré‐Antrais (LAMOFOPA), Universidade Estadual do Ceará (UECE) Fortaleza CE Brazil

**Keywords:** cryopreservation, dimethyl sulfoxide, ethylene glycol, glycerol, testicles

## Abstract

Testicular vitrification requires the use of high concentrations of cryoprotectants, which can cause damage to samples due to their toxicity. The combination of these substances comes up as a way to mitigate this problem. Thus, the aim of this study was to evaluate three cryoprotectant combinations in the testicular vitrification of dogs. Ten testicular pairs from adult dogs were used, from which 12 fragments of each pair were obtained, distributed among the fresh control group (CTR) and the experimental groups according to the cryoprotectant combinations tested: dimethyl sulfoxide (DMSO)/ethylene glycol (EG), DMSO/glycerol (GLY), and EG/GLY. The fragments were vitrified using the solid surface vitrification method (SSV), at a final concentration of 5.6 mol/L of the combined cryoprotectants. Subsequently, they were warmed up and processed for histomorphological morphometric evaluations and determination of mitochondrial activity with Rhodamine 123. Considering the morphological evaluation, the DMSO/EG group showed results similar to CTR, with good scores for nuclear integrity and cell organisation in the seminiferous tubules (*p* > 0.05). In contrast, the EG/GLY group presented greater nuclear condensation. It was difficult to visualise and distinguish between spermatogonia and Sertoli cells (*p* < 0.05). The DMSO/GLY group also showed distinct levels between spermatogonia and Sertoli cells, as well as nuclear condensation, which statistically differed from CTR (*p* < 0.05). Also, it was observed a random distribution of the remaining cells in the seminiferous tubules of the EG/GLY and DMSO/GLY groups. The three tested groups showed basement membrane retraction and a reduction of approximately 11.6% in the average diameter of the seminiferous tubules (*p* < 0.05). Vitrification did not influence the mitochondrial activity of the samples, regardless of the combination of cryoprotectants used (*p* > 0.05). It was concluded that the DMSO/EG combination best contributed to the maintenance of the testicular histomorphological structure of dogs after vitrification.

## 1 Introduction

Testicular cryopreservation is a recent technology in domestic (Lee et al. 2016; Lima et al. 2018) and wild mammals (Thuwanut et al. 2013; Pothana et al. 2015; Andrae et al. 2021) which allows the use of germplasm in assisted reproductive techniques, such as intracytoplasmic sperm injection (Yokonishi et al. 2013) and in vitro fertilisation (Higaki et al. 2018). It represents one of the few alternatives to preserve male fertility potential when it is not possible to obtain sperm, as occurs in pre‐pubertal individuals or animals that spend most of the year outside their reproductive season, as some species of wild canids (Comizzoli et al. 2018; Andrae et al. 2021).

Among the cryopreservation methods for testicular samples, vitrification stands out as a promising alternative due to its lower cost and fast execution compared to slow freezing (Fernandes et al. 2021). To perform this technique, it is necessary to use solutions with high concentrations of cryoprotectants and fast temperature reduction (20,000–40,000°C/min). Due to high concentrations of cryoprotectants, solutions become more viscous as the temperature decreases, thus forming an amorphous solid (Picazo et al. 2022). It reduces the formation of ice crystals and, consequently, the harmful effects they could have on cell viability (Portillo et al. 2006).

Dimethyl sulfoxide (DMSO), ethylene glycol (EG) and glycerol (GLY) stand out among the penetrating cryoprotectants used in testicular cryopreservation protocols (Lima et al. 2018). DMSO has shown satisfactory results in preserving the integrity of samples (Gossens et al. 2008). It interacts with the lipid membrane and induces the formation of pores for the passage of water, causing cellular dehydration and, consequently, reducing the formation of ice crystals inside cells during cryopreservation (Gurtovenko and Anwar 2007). On the other hand, EG can permeate the cell membrane more quickly (Cooper et al. 2008), resulting in lower osmotic stress and preventing the formation of ice crystals during cryopreservation (Weng et al. 2011). GLY is a highly permeable polyhydric alcohol that binds to hydrogen ions in the water molecule and reduces the speed of osmotic dehydration, minimising cellular damage. Furthermore, it lowers the freezing point, reduces electrolyte concentrations in the unfrozen fraction of the sample, and minimises the formation of intracellular ice crystals (Doebbler 1966; Watson 2000).

However, the use of high concentrations of cryoprotectants may generate toxic effects, resulting in damage to cells and tissues (Buarpung et al. 2013). To mitigate this problem, the combination of cryoprotectants is used to lower these concentrations and, consequently, reduce the toxicity of these agents (Poels et al. 2012; Pukazhenthi et al. 2015). This approach has already been tested in the vitrification of testicular samples from pre‐pubertal cats (Lima et al. 2016, 2018), collared peccaries (Da Silva et al. 2019), adult grey wolves (Andrae et al. 2021) and pre‐pubertal dogs (Teixeira et al. 2021). However, testicles from pubescent individuals are more sensitive to manipulation due to the complex metabolism of different cell types present in the seminiferous tubules (Mota et al. 2012; Fayomi et al. 2019). Therefore, this study aimed to evaluate the effect of different combinations of cryoprotectants during the vitrification of the testicles of adult dogs.

## 2 Materials and Methods

The Animal Ethics Committee of the State University of Ceará (UECE) approved this study, protocol no. 11869984/2022.

### 2.1 Testis Collection and Experimental Design

Ten medium‐sized mongrel male dogs (*n* = 10) were evaluated in this study. They were clinically healthy, aged between 1 and 5 years, with no history of reproductive diseases and no macroscopic changes in the testicles. The dogs underwent elective orchiectomy at the Professor Sylvio Barbosa Cardoso Veterinary Hospital, located at the State University of Ceará in Fortaleza/CE, Brazil.

After orchiectomy, each testicular pair was transported from the veterinary hospital to the laboratory in a Falcon tube containing saline solution (0.9% NaCl) at room temperature (~ 22°C), within a maximum period of 1 h. In the laboratory, the testes were separated from the surrounding tissues in Ham's F10 medium (Sigma Aldrich, N6908), and fragments measuring approximately 3 x 3 x 1 mm were obtained using scalpel blades.

**FIGURE 1:**
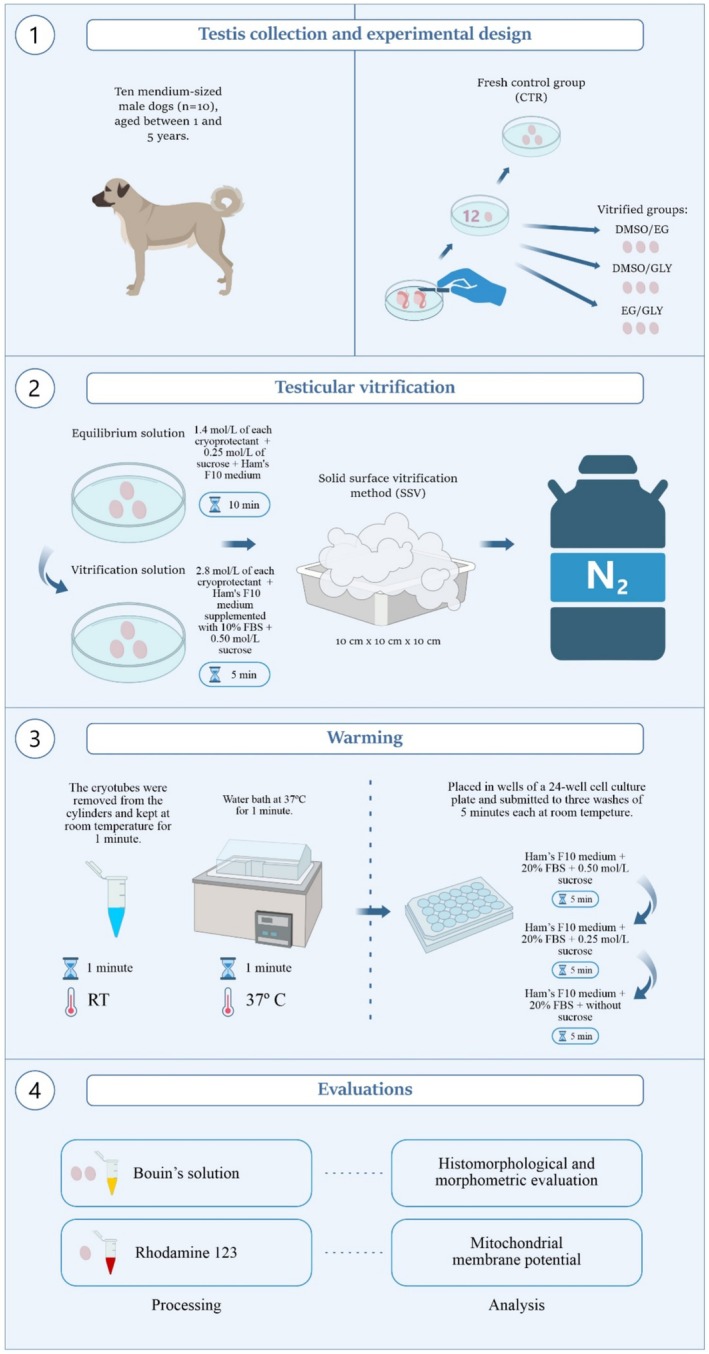
Experimental design. Distribution of experimental groups according to cryoprotectant combinations and evaluation techniques.

From each testicular pair, 12 fragments were randomly collected, distributed as follows: three fragments were allocated to the fresh control group (CTR), two of which were fixed for histological analysis and one sent for fluorescence microscopy immediately after fragmentation. The remaining fragments were randomly divided among the three vitrified groups, using three different cryoprotectant combinations: DMSO/EG, DMSO/GLY and EG/GLY (all cryoprotectants were obtained from Neon Comercial) (Figure 1).

### 2.2 Testicular Vitrification

The fragments were initially immersed in an equilibrium solution (total volume: 1.8 mL) composed of 1.4 mol/L of each cryoprotectant (DMSO/EG, DMSO/GLY and EG/GLY), 0.25 mol/L of sucrose, and Ham's F10 medium for 10 min at room temperature. Then, the fragments were exposed to the vitrification solution (total volume: 1.8 mL), which contained 2.8 mol/L of each cryoprotectant (final concentration of 5.6 mol/L) in Ham's F10 medium supplemented with 10% foetal bovine serum (FBS) and 0.50 mol/L sucrose for 5 min at room temperature (Lima et al. 2018). After this process, the testicular fragments were submitted to the solid surface vitrification method (SSV). They were transferred to a metal cube made of aluminium foil (10 cm × 10 cm × 10 cm) with the aid of a needle, without perforating the fragment, and partially immersed in liquid nitrogen. Then, the fragments were relocated to pre‐cooled 2 mL cryotubes and stored in liquid nitrogen cylinders for at least one week (Abrishami et al. 2010; Baert et al. 2012; Lima et al. 2016).

### 2.3 Warming

After the storage period, the cryotubes were removed from the cylinders and kept at room temperature for one minute. They were then immersed in a water bath at 37°C for another minute. In order to remove the cryoprotectants, the fragments were cleared from the cryotubes, placed in wells of a 24‐well cell culture plate and submitted to three washes of 5 min each at room temperature. The procedure was performed at Ham's F10 medium supplemented with 20% FBS and decreasing concentrations of sucrose (0.50 mol/L, 0.25 mol/L and without sucrose) (Lima et al. 2016).

### 2.4 Histomorphological and Morphometric Evaluation

Testicular fragments of the CTR and those obtained after removal of the cryoprotectants were fixed in Bouin's solution for 24 h and then transferred to 70% alcohol solution. Following histological processing, the fragments were dehydrated using increasing concentrations of alcohol, embedded in paraffin, and sectioned into 5 μm thick sections. The slides were stained with haematoxylin–eosin (Baert et al. 2012; Teixeira et al. 2021).

The slides were analysed using a microscope equipped for digital photomicrography (Nikon Eclipse Ci‐L Microscope; Nikon DS‐Fi3 Camera, USB 3.0, 5.9‐megapixel CMOS image sensor). In the histomorphological evaluation, 30 seminiferous tubules from each sample were evaluated based on seven parameters, using a scale of 1 to 3 (Table 1), where 1 corresponds to normal morphology, 2 to discrete or moderate changes, and 3 to severe changes (Lima et al. 2016); (Da Silva et al. 2019).

**TABLE 1:** Histomorphological parameters of seminiferous tubules from canine testicles.

Parameter	Score 1	Score 2	Score 3
CSBM	None	Separation < 75%	Separation > 75%
BMR	None	Little retraction	Exacerbated retraction
DSS	Clear	Not well defined	Impossible distinction
Nuclear visualisation	Easy	Difficult	Impossible
Nuclear condensation	None	< 50% of cells with nuclear condensation	> 50% of cells with nuclear condensation
Tubular cell loss	None	< 75% cell types lost	> 75% cell types lost
Tubular structure	Intact	All cell types present although slightly disordered structure	Random distribution of remaining cells

Abbreviations: BMR, basal membrane retraction; CSBM, cellular separation from the basal membrane; DSS, distinction between spermatogonia and Sertoli cells.

*Source:* Adapted from Lima et al. (2016) and Da Silva et al. (2019).

The morphometric evaluation was carried out using the NIS Elements software (Nikon, Nikon Instruments Inc.). This program allowed the calculation of the average of the two largest perpendicular diameters of 20 seminiferous tubules in cross‐sections of each sample (Teixeira et al. 2021).

### 2.5 Assessment of Mitochondrial Membrane Potential

The testicular fragments were macerated with the aid of a scalpel blade. Then, they were incubated in a dark chamber in Ham's F10 medium supplemented with 10% FBS and 10 μg/mL of Rhodamine 123 solution (Invitrogen, Thermo Fisher Scientific, R302), for 15 min at 37°C. Subsequently, the samples were washed twice in phosphate‐buffered saline for 1 min. Slides were then prepared with the samples exposed to the ProLong Gold Antifade Mountant (Invitrogen, Thermo Fisher Scientific, P36930). The images were obtained using an epifluorescence microscope (Olympus BX41—Olympus Corporation). NIS‐Elements software allowed the measurement of fluorescence at 40x magnification.

The microscope camera configuration was kept constant throughout the experiments, as well as the area evaluated for all samples. Relative intensity was determined by subtracting the fluorescence emission of the tissue with Rhodamine 123 from the back (dark) of each slide. The 10 areas with the highest fluorescent intensity in each sample were analysed, and the mitochondrial membrane potential was evaluated based on the intensity of Rhodamine 123. The potential classification was directly proportional to this intensity (Fernandes et al. 2021).

### 2.6 Statistical Analysis

Data were expressed as mean and standard error and analysed using Python 3.9, Scipy and Scikit‐learn. Data distribution was analysed using the Shapiro–Wilk and Bartlett tests. Analysis of variance (ANOVA) was used to compare the mean proportions among groups with normal distribution, followed by the Tukey test. When the data did not present a normal distribution, the Kruskal‐Wallis test was used, followed by the Dunn test. Differences were considered significant when *p* < 0.05.

## 3 Results

**FIGURE 2:**
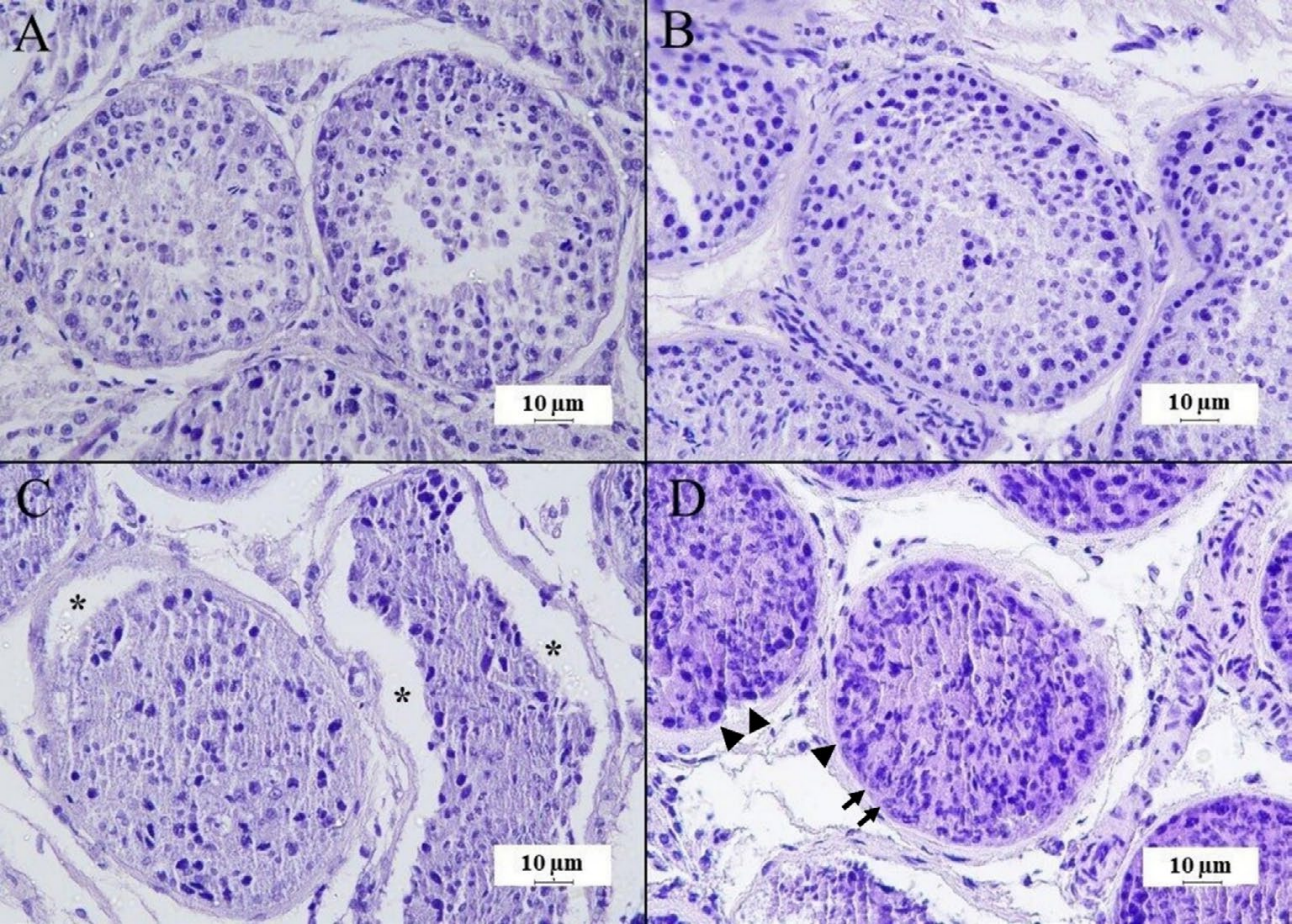
Histomorphological evaluation of canine testicular fragments. (A) Fresh control group (CTR); (B) DMSO/EG group showing good integrity; (C) DMSO/GLY group with cellular loss and basement membrane separation (asterisks); (D) EG/GLY group showing disorganised tubular structure and difficult distinction between spermatogonia (arrowheads) and Sertoli cells (arrows). (HE, 400x, scale: 10 μm).

In the histomorphological evaluation, DMSO/EG presented results closer to the CTR, with good scores in the three parameters observed in the evaluation of nuclear integrity (*p* > 0.05). Furthermore, good organisation of cells within the seminiferous tubules was also observed (*p* > 0.05) (Figure 2B, Table 2).

**TABLE 2:** Histomorphological analysis of canine testicular fragments. Fresh control group (CTR) and groups vitrified with DMSO/EG, DMSO/GLY, and EG/GLY.

PARAMETERS	CTR	DMSO/EG	DMSO/GLY	EG/GLY
CSBM	1.12 ± 0.11^a^	1.25 ± 0.15^ab^	1.38 ± 0.20^b^	1.32 ± 0.21^ab^
BMR	1.01 ± 0.02^a^	1.18 ± 0.15^b^	1.24 ± 0.23^b^	1.30 ± 0.22^b^
DSS	1.10 ± 0.10^a^	1.26 ± 0.17^ab^	1.48 ± 0.18^bc^	1.70 ± 0.26^c^
Nuclear visualisation	1.13 ± 0.12^a^	1.15 ± 0.11^a^	1.28 ± 0.21^ab^	1.39 ± 0.23^b^
Nuclear condensation	1.37 ± 0.32^a^	1.42 ± 0.17^a^	1.90 ± 0.24^b^	1.95 ± 0.33^b^
Tubular cell loss	1.07 ± 0.07^a^	1.09 ± 0.05^ab^	1.19 ± 0.10^b^	1.10 ± 0.09^ab^
Tubular structure	1.37 ± 0.19^a^	1.70 ± 0.39^a^	2.63 ± 0.19^b^	2.52 ± 0.26^b^

*Note:* Lowercase letters superscript differently on the same line indicate statistical differences among groups (*p* < 0.05).

Abbreviations: BMR, basal membrane retraction; CSBM, cellular separation from the basal membrane; DSS, distinction between spermatogonia and sertoli cells.

Regarding nuclear integrity, the EG/GLY association had the most negative impact on the nuclei of germ cells. This group presented a difficult distinction between spermatogonia and Sertoli cells, greater difficulty in nuclear visualisation and a greater presence of condensed nuclei compared to the control group (*p* < 0.05) (Figure 2D). DMSO/GLY also presented distinction scores between spermatogonial and Sertoli cells and nuclear condensation that differed statistically from the CTR (*p* < 0.05) (Figure 2C). In addition, it showed a high level of separation of the basement membrane and cell loss among the experimental groups (*p* < 0.05). Furthermore, the three vitrified groups showed basement membrane retraction (*p* < 0.05). The EG/GLY and DMSO/GLY groups presented a random distribution of the remaining cells in the seminiferous tubules (*p* < 0.05) (Table 2, Figure 2C,D).

**FIGURE 3:**
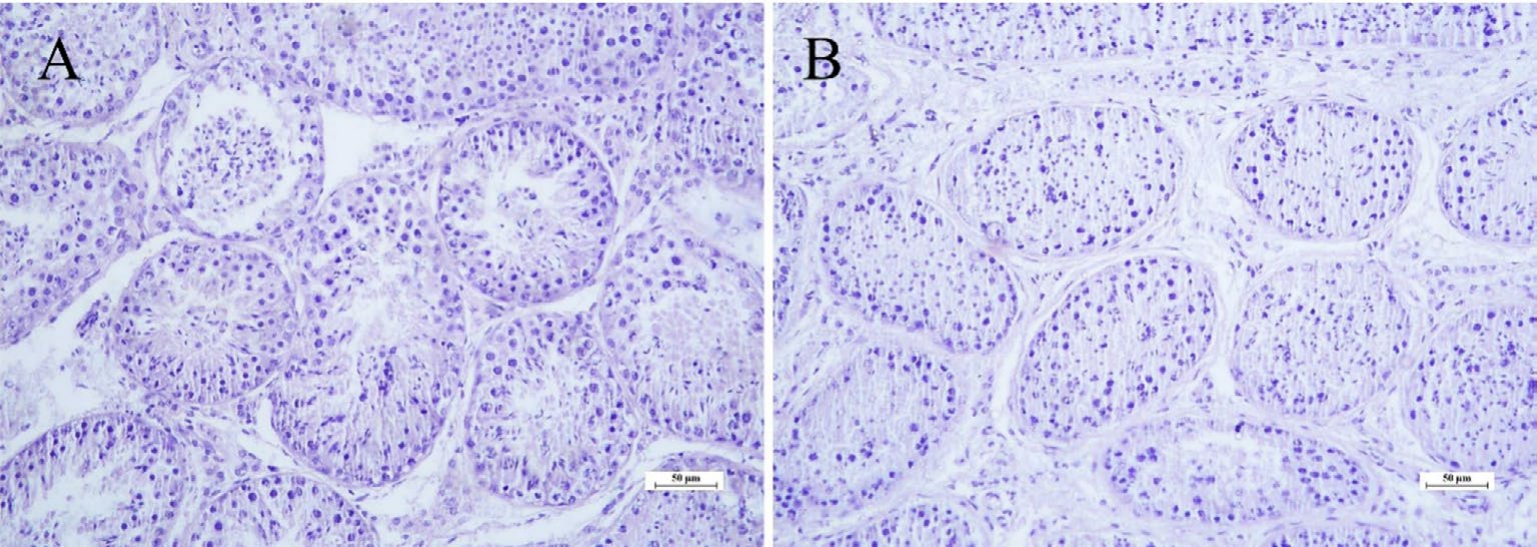
Photomicrograph illustrating the reduction in tubular diameter after vitrification. (A) Fresh control group; (B) DMSO/EG group. (HE, 200x, scale: 50 μm).

**FIGURE 4:**
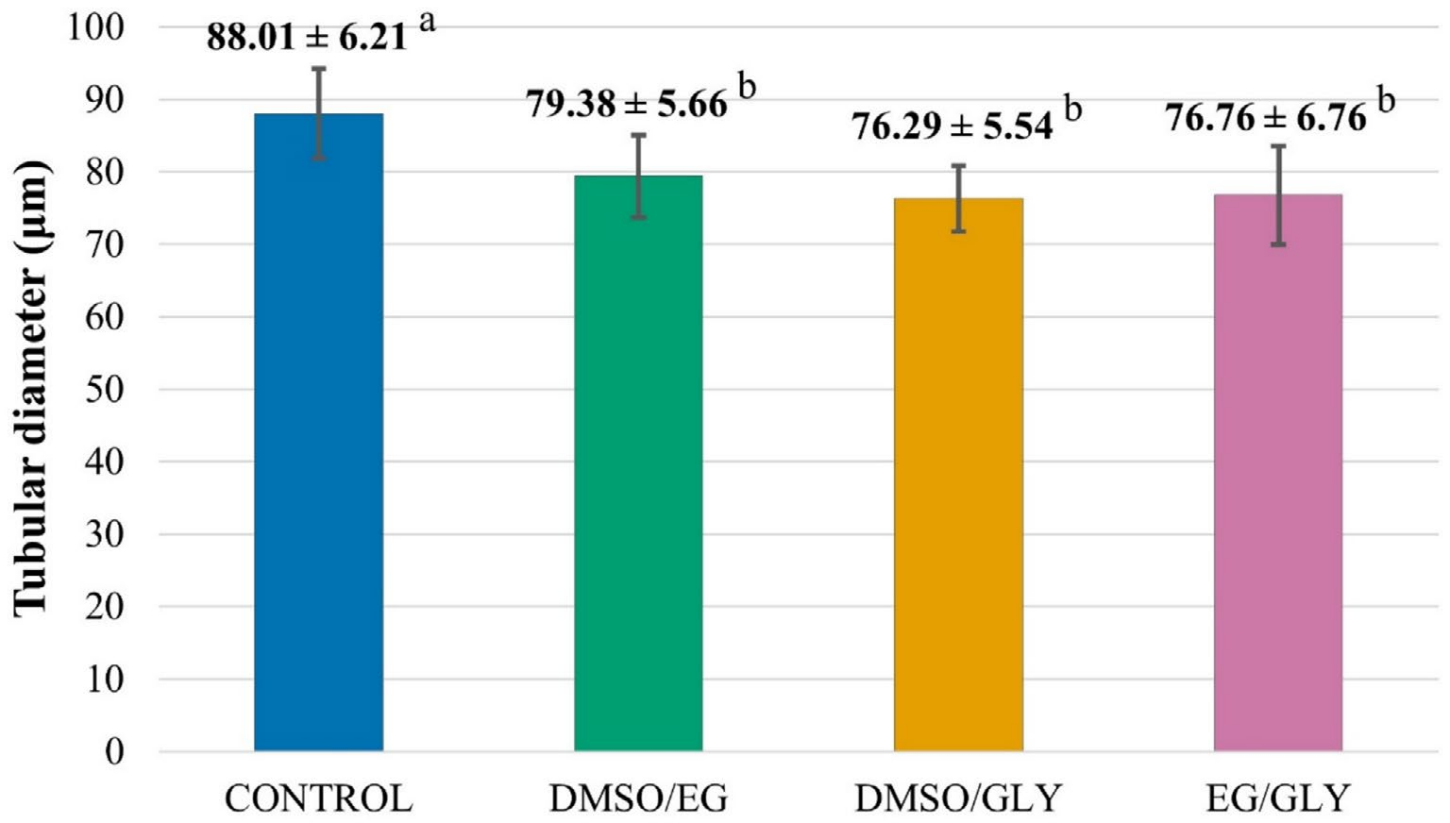
Mean ± SD of the diameter of canine seminiferous tubules. Fresh control group (CTR) and vitrified groups with different cryoprotectant combinations: DMSO/EG, DMSO/GLY, and EG/GLY, respectively. Lowercase letters superscript differently on the same line indicate statistical differences among groups (*p* < 0.05).

In the morphometric evaluation, the three vitrified groups showed no differences among them (*p* > 0.05). However, they showed a reduction of approximately 11.6% in the diameter of the seminiferous tubules when compared to the CTR (*p* < 0.05) (Figure 3A,B, Figure 4).

**FIGURE 5:**
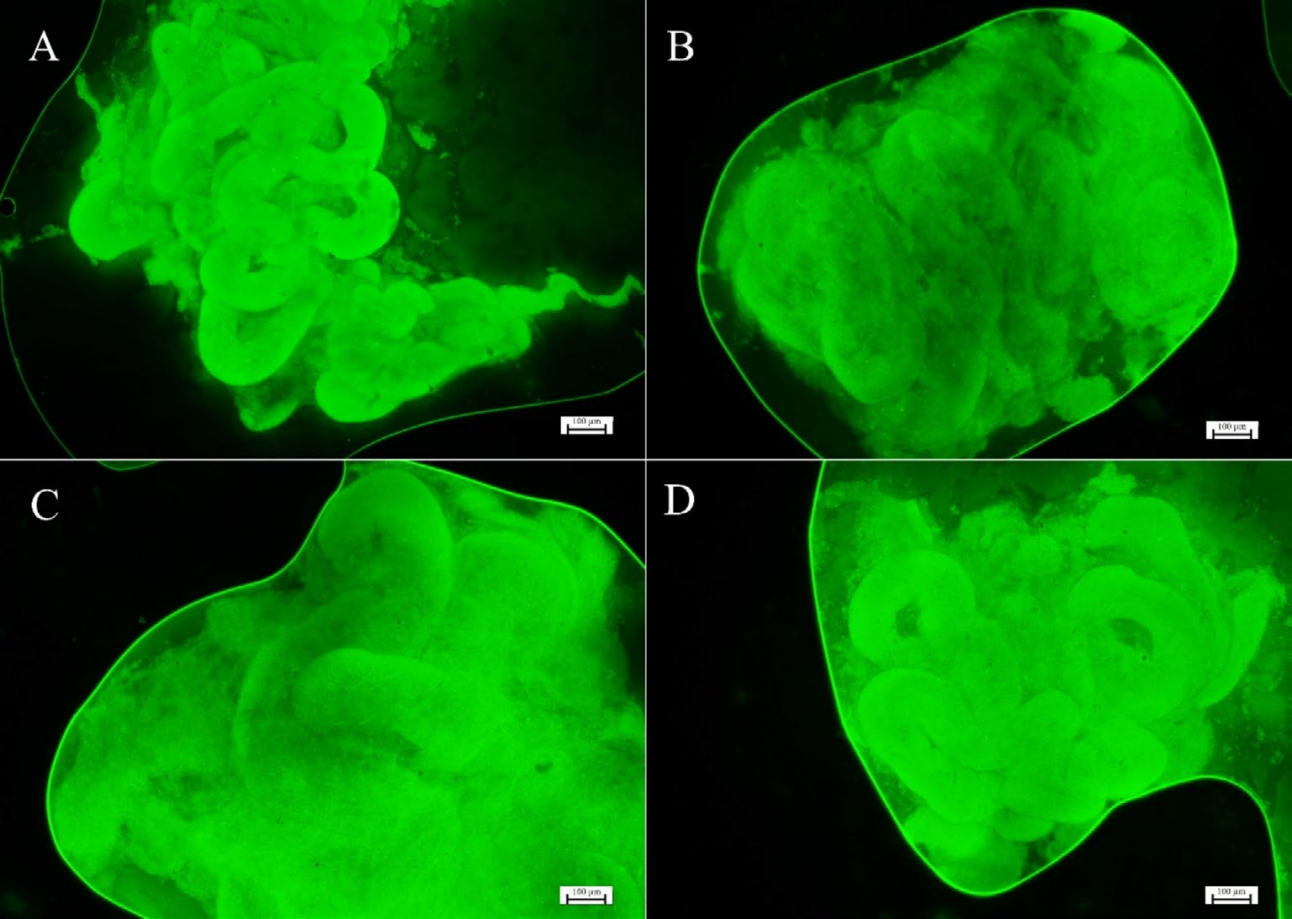
Photomicrographs of testicular fragments demonstrating mitochondrial activity under fluorescent microscopic analysis with Rhodamine 123. (A) Fresh control group (CTR); (B) DMSO/EG; (C) DMSO/GLY; (D) EG/GLY. (40x; scale: 100 μm).

**FIGURE 6:**
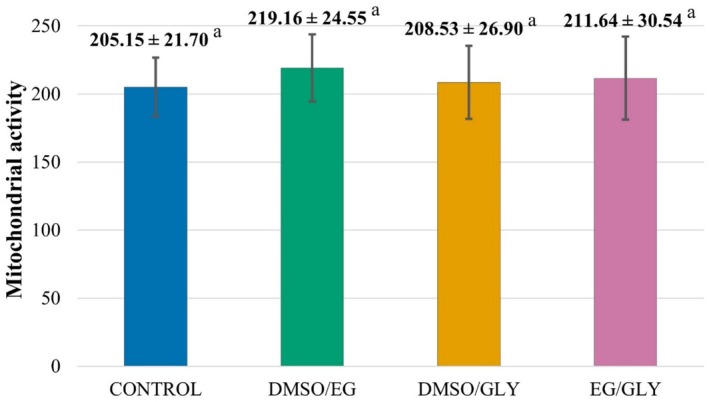
Evaluation of the viability of canine testicular fragments, fresh control group (CTR) and vitrified groups with different cryoprotectant combinations: DMSO/EG, DMSO/GLY, and EG/GLY. Lowercase letters superscript differently on the same line indicate statistical differences between groups (*p* < 0.05).

Regarding the evaluation of the mitochondrial membrane potential, the vitrified samples did not differ statistically from each other, nor from the fresh samples (*p* > 0.05), demonstrating that the different combinations of cryoprotectants did not affect mitochondrial activity (Figure 5A–D and Figure 6).

## 4 Discussion

In cryopreservation, combining cryoprotectants is a strategy to minimise individual toxicity while maintaining cell viability. Therefore, in this study, three cryoprotectant combinations were tested during SSV at the same concentrations (final concentration of 5.6 mol/L) used by Lima et al. (2016) and Teixeira et al. (2021).

In the vitrification of testicular fragments from pre‐pubertal cats using the SSV method, the combination of DMSO/GLY was effective in maintaining the morphological characteristics of the samples (Lima et al. 2016). On the other hand, in one of the few studies that investigated canine testicular vitrification, it was observed that testicular fragments from pre‐pubertal dogs showed better structure preservation when vitrified with EG/GLY or DMSO/EG (Teixeira et al. 2021). In our study, the DMSO/EG combination showed better performance in preserving the integrity of the germ cell nuclei and the structural organisation of the seminiferous tubules of dogs after vitrification. This discrepancy underscores the importance of developing species‐specific cryopreservation protocols, considering particularities such as the animal's age and unique physiological characteristics.

In this study, DMSO/EG presented a satisfactory score for the basement membrane cell separation parameter (1.25 ± 0.15), which indicates preservation of the integrity of the epithelial lining of the seminiferous tubules. This result differs from what was observed in a previous study that performed the vitrification of testicles from adult grey wolves (Andrae et al. 2021). The same combination of cryoprotectants (15% DMSO/15% EG) resulted in greater separation of the basement membrane (score 2, 25 to 75% separation). In this way, there was greater impairment of the tubules. This difference may be attributed to the vitrification method adopted, as this study used SSV, while the comparative study used needle immersion vitrification (NIV). Considering that both species belong to the same genus, the results suggest that SSV may be a more effective method than NIV regarding preservation of the integrity of canine testicular samples.

In the vitrification of pubescent collared peccary testicles, it was observed that the DMSO/EG association provided better preservation of the testicular structure (tubular lumen, cell junctions and cell membrane integrity), as well as the proliferative capacity of spermatogonia and Sertoli cells (Da Silva et al. 2019), when compared to the isolated use of each cryoprotectant. The authors also observed that vitrification did not affect nuclear visualisation and condensation of testicular cell nuclei, regardless of the intracellular cryoprotectants used. In this present study, these two parameters remained similar to those of CTR and DMSO/EG (*p* > 0.05), while EG/GLY and DMSO/GLY showed changes (*p* < 0.05), demonstrating that the use of GLY can negatively affect the structure of dog germ cell nuclei during vitrification.

GLY was more effective for spermatozoa and spermatids, while DMSO and EG were more effective for spermatogonia and spermatocytes (Unni et al. 2012; Buarpung et al. 2013). Corroborating the authors mentioned above, it was possible to observe greater difficulty in distinguishing Sertoli cells from spermatogonia in the fragments that were submitted to vitrification using GLY as one of the cryoprotectants, demonstrating its lower capacity for cryoprotection of spermatogonia.

In the morphometric analysis, a reduction in the diameter of the seminiferous tubules was observed in all groups submitted to vitrification, as was observed in the vitrification of testicular samples from pre‐pubertal cats (Fernandes et al. 2021). A possible explanation for this could be the thermal stress to which the tubules are submitted during vitrification. According to other studies, there is a possibility of recovery of the tubular diameter after cultivation and xenograft of the fragments (Wyns et al. 2007; Ma et al. 2022).

There are still major challenges in maintaining intact testicular samples and cells of the spermatogenic lineage viable and functional after the vitrification process (Yokonishi et al. 2013), especially in samples from adult individuals, due to their cellular diversity and vulnerability to ex vivo conditions (Sato et al. 2015). Although vitrification can cause damage to germ cell metabolism, including impacts on mitochondrial activity (Lima et al. 2018), this study demonstrated, through fluorescence microscopy using Rhodamine 123, the reestablishment of mitochondrial function after post‐vitrification warming.

Similar results were observed in studies with vitrification of pre‐pubertal cat testicles after warming (Fernandes et al. 2021) and after short‐term in vitro culture (Lima et al. 2018), in which an increase in mitochondrial activity was even observed. These findings reinforce the potential of vitrification as a viable tool for the conservation of testicular samples, highlighting the importance of the warming process in maintaining mitochondrial function and, consequently, the cellular metabolism of the samples.

## 5 Conclusion

The combination of dimethyl sulfoxide (DMSO) and ethylene glycol (EG) has been identified as the most effective in preserving the histomorphological structure of canine testicular fragments following the solid‐surface vitrification (SSV) procedure. This finding aligns with previous research on testicular cryopreservation in other species. Based on the results, this study aims to stimulate further research using canine testicular samples and contribute to the development of assisted reproductive technologies aimed at conserving endangered canid species.

## Author Contributions

Jéssyka Noronha, Juliana Fernandes, Julia Colares, and Gisele Guimarães performed the experiments; Jéssyka Noronha and Juliana Fernandes carried out the laboratory analyses; Francisco Rodrigues, Jéssyka Noronha, and Juliana Fernandes operated the histological processing; Lúcia Silva was responsible for project supervision and coordination; Jéssyka Noronha and Lúcia Silva wrote the manuscript; and Herlon Silva and Lúcia Silva were responsible for the final review of the manuscript.

## Conflicts of Interest

The authors declare no conflicts of interest.

## Data Availability

The data supporting the findings of this study are available upon request from the corresponding author.
